# Stigmasterol alleviates neuropathic pain by reducing Schwann cell‐macrophage cascade in DRG by modulating IL‐34/CSF1R

**DOI:** 10.1111/cns.14657

**Published:** 2024-04-04

**Authors:** Waimei Si, Zhenni Chen, Jing Bei, Shiquan Chang, Yachun Zheng, Li Gao, Guoping Zhao, Xin Li, Di Zhang

**Affiliations:** ^1^ School of Traditional Chinese Medicine Jinan University Guangzhou China

**Keywords:** CCI, CSF1R, DRG, IL‐34, single‐cell sequencing, stigmasterol

## Abstract

**Aims:**

This study aimed to investigate the potential therapeutic applications of stigmasterol for treating neuropathic pain.

**Methods:**

Related mechanisms were investigated by DRG single‐cell sequencing analysis and the use of specific inhibitors in cellular experiments. In animal experiments, 32 male Sprague–Dawley rats were randomly divided into the sham operation group, CCI group, ibuprofen group, and stigmasterol group. We performed behavioral tests, ELISA, H&E staining and immunohistochemistry, and western blotting.

**Results:**

Cell communication analysis by single‐cell sequencing reveals that after peripheral nerve injury, Schwann cells secrete IL‐34 to act on CSF1R in macrophages. After peripheral nerve injury, the mRNA expression levels of CSF1R pathway and NLRP3 inflammasome in macrophages were increased in DRG. In vitro studies demonstrated that stigmasterol can reduce the secretion of IL‐34 in LPS‐induced RSC96 Schwann cells; stigmasterol treatment of LPS‐induced Schwann cell‐conditioned medium (L‐S‐CM) does not induce the proliferation and migration of RAW264.7 macrophages; L‐S‐CM reduces CSF1R signaling pathway (CSF1R, P38MAPK, and NFκB) activation, NLRP3 inflammasome activation, and ROS production. In vivo experiments have verified that stigmasterol can reduce thermal and cold hyperalgesia in rat chronic compressive nerve injury (CCI) model; stigmasterol can reduce IL‐1β, IL‐6, TNF‐α, CCL2, SP, and PGE2 in serum of CCI rats; immunohistochemistry and western blot confirmed that stigmasterol can reduce the levels of IL‐34/CSF1R signaling pathway and NLRP3 inflammasome in DRG of CCI rats.

**Conclusion:**

Stigmasterol alleviates neuropathic pain by reducing Schwann cell‐macrophage cascade in DRG by modulating IL‐34/CSF1R axis.

## Graphical abstract

Stigmasterol reduces the cascade reaction of Schwann cells and macrophages by regulating the IL34/CSF1R axis, and reduces the peripheral sensitization caused by neuroinflammation in the dorsal root ganglia (DRG), thereby alleviating neuropathic pain.

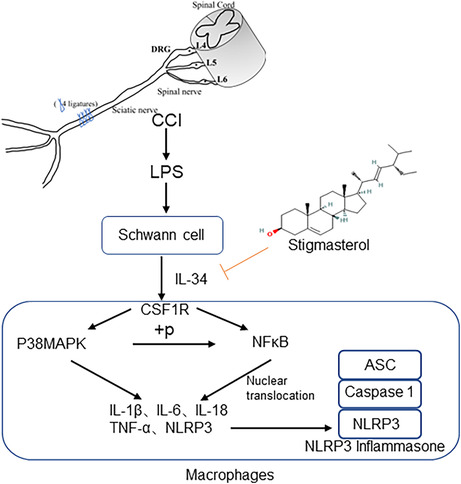

## 1 INTRODUCTION

Neuropathic pain (NP) is caused by damage or disease of the peripheral or central somatosensory nervous system, and its main clinical manifestations are increased pain sensitivity caused by decreased pain threshold.^1^ Globally, a considerable population of about 7%–10% suffers from NP often leading to a decline in their physical and mental well‐being.^2^ Recent studies have highlighted the beneficial effects of NSAIDs such as ibuprofen, which is a widely used short half‐life yet long‐lasting drug for the treatment of NP.^3^ Studies have demonstrated that NSAIDs, like ibuprofen, can effectively attenuate sensory hypersensitivity in animal models of neuropathic pain.^4^ However, the side effects of NSAIDs, such as adverse gastrointestinal reactions and abuse, limit their long‐term use. Therefore, innovative medications are critically needed to treat NP.

Peripheral sensitization is one of the critical mechanisms leading to NP, which involves interactions between neurons, immune cells, and Schwann cells.^5^ Schwann cells in the dorsal root ganglion (DRG) undergo reprogramming to initiate the release of chemokines and cytokines to promote macrophage recruitment and infiltration after peripheral nerve injury (PNI).^6^ The inflammatory mediators produced by the macrophages in DRG mediate NP and have been the subject of intense research.^7^ The CSF signaling pathway is associated with the proliferation, activation, and migration of macrophages that contribute to NP.^8^ The CSF1R, a type III tyrosine kinase transmembrane receptor, is activated by two ligands, interleukin (IL)‐34 and colony‐stimulating factor 1 (CSF1), to maintain tissue macrophage homeostasis.^9^ The IL‐34/CSF‐1R signaling pathway plays a crucial role in determining macrophage development and proliferation in the peripheral nervous system (PNS).^10^ Binding of IL‐34 to CSF‐1R triggers the activation of many signaling pathways, including but not limited to extracellular signal‐regulated protein kinases 1 and 2 (ERK1/2), nuclear factor kappa‐B (NF‐κB), focal adhesion kinase (FAK), P38 mitogen‐activated kinase (P38MAPK), and Scr kinase family.^8^ Therefore, we suggest that the inhibition of IL‐34/CSF1R signaling may be a promising strategy for relieving neuropathic pain.

Stigmasterol is a phytosterol with anti‐inflammatory properties and neuroprotective properties.^11^, ^12^ Studies have shown that stigmasterol has the potential to reduce acute and chronic pain in rat and mouse models.^13^, ^14^ Stigmasterol has also been found to attenuate the expression of various inflammatory mediators in macrophages, including NO, TNF‐α, COX‐2, and iNOS.^15^

This study aims to investigate the therapeutic effect of stigmasterol on NP and its underlying mechanism. We investigated the underlying mechanisms at the cellular level through cellular communication analysis and in vitro experiments. The analgesic effect of stigmasterol on the CCI model was observed through behavioral, ELISA, pathological, and immunohistochemical methods.

## 2 MATERIALS AND METHODS

### 2.1 Reagents

Stigmasterol (S31030, 95% purity) for the in vivo experiments was purchased from Shanghai yuanye Bio‐Technology Co., Ltd; stigmasterol (AB0177‐0020, HPLC ≥ 98%) for the in vitro experiments was purchased from Chengdu Alfa Biotechnology Co., Ltd. Ibuprofen (Drug approval number: H20066822) for the in vivo experiments was obtained from Guangzhou Overseas Hospital, First Affiliated Hospital of Jinan University. LPS (L2880) was purchased from Sigma‐Aldrich (Shanghai) Trading Co Ltd. GW2580 (an inhibitor of CSF1R), SB203580 (an inhibitor of P38MAPK), and CP‐456773 (an inhibitor of NLRP3) were purchased from Shanghai Yuanye Biotechnology Co., Ltd. SYBR Green Premix qPCR Kit and Evo M‐MLV RT Mix Kit (AG11701 and AG11012, respectively) were purchased from Accurate Biotechnology Co., Ltd. CCK8‐solution (ST1008) was purchased from Shanghai Shangbao Biotechnology Co., Ltd. A Cell Membrane Protein and Cytoplasmic Protein Extraction Kit (BL671A) was purchased from Biosharp Biotechnology Co., Ltd. (China). IL‐1β (MM‐0047R1), IL‐6 (MM‐0190R1), TNF‐α (MM‐0180R1), CCL2(MM‐0848R1), SP (MM‐0444R1), and PGE2 (MM‐0068R1) ELISA kits were purchased from American Cotton Industry Co., Ltd. (China). IL‐34 (QZ‐14731) was purchased from Quanzhou jiubang Biotechnology Co., Ltd. The following antibodies were used: IL‐34 (31957, Signalway Antibody), CSF1R (38,540, Signalway Antibody), anti‐Iba‐1 (A5595, BIMAKE) (GB12105, Servicebio), anti‐IL‐1β (WL00891, BIODOG), anti‐IL‐18 (M027287, Abmart), anti‐β‐actin (GB11001, Servicebio), anti‐NFκB (8242S, CST), anti‐p‐NFκB (3033, CST), anti‐P38MAPK (8690S, CST), anti‐phospho‐P38MAPK (4511S, CST), Caspase 1 (381,016, ZENBIO) and ASC (340097, ZENBIO), NLRP3(WL02635, BIODOG), and goat anti‐rabbit IgG (H + L) HRP (E030120‐2, EARTH).

### 2.2 Single‐cell data quality control and cell annotation

Single‐cell sequencing data of the mouse L4 dorsal root ganglion (DRG) in the GSE158892 dataset was downloaded from the GEO database (https://www.ncbi.nlm.nih.gov/geo/). Two L4 DRG samples from Naive (noninjured mice) and one L4 DRG sample from SNC (sciatic nerve‐injured mice) were selected for subsequent data analysis, constituting a total of 13,010 cells. To ensure the reliability of the subsequent analyses, low‐quality cell data were removed using the “Seurat” R package for cell quality control and cell type identification. The parameters were set as UMI > 200, nFeatures <5000, and mtRNA <10% to filter out low‐quality cells. Next, the data were normalized with the parameter settings of normalization method = “LogNormalize” and scale.factor = “10,000.” Hypervariable genes were calculated, and principal component analysis (PCA) was performed. The data were then clustered and visualized using the t‐distributed stochastic neighbor embedding (t‐SNE) algorithm.

The Naive group and the SNC group data were merged using the “Harmony” R package, and batch effects were removed. After the merge, cell annotation was performed using the “FindAllMarker” function in “Seurat” to obtain the top 10 marker genes of each cell type. The built‐in dataset “ImmGenData” in the “SingleR” package was used to annotate the clustering results. Cell subpopulations were then annotated based on the marker genes and published literature.

### 2.3 Analysis of DRG cell communication in sciatic nerve injury

The study utilized the “Cellchat” R package to analyze single‐cell transcriptional sequencing data. After quality control and cell annotation, distinct cell types in DRGs were identified. The Paracrine/Autocrine Signaling Interaction dataset of CellChatDB served as a reference database to understand cell–cell interactions in DRG. The communication probability was then computed using a truncated mean of 20% (function computeCommunProb, type = “truncatedMean,” trim = 0.2). The analysis inferred intercellular communication, which allowed for the aggregating of intercellular communication networks using default parameters. The number of interactions was visualized to reveal aggregated intercellular communication networks and signals sent from each cell cluster. Schwann cells and macrophages were selected for visualizing ligand–receptor interactions. Finally, the IL‐34/CSF1R signaling pathway was selected for visualization.

Additionally, the Seurat “FindAllMarkers” function was used to analyze the differences between macrophages in Naive mouse DRG samples and SNC mouse DRG samples. Thresholds of Foldchange >1.0 and *p* < 0.05 were set to obtain the differential genes of the two groups. The expression differences of CSF1R(Csf1R), P38 MAPK (Mapk 14), and NF‐ΚB p65 (Rela) in the CSF1R signaling pathway between the two groups of macrophages were determined. Finally, the expression differences of macrophage activation marker Iba‐1 (Aif1) and NLRP3 inflammasome (Nlrp3, Pycard, and Casp1) were calculated, and adjusted *p*‐values were determined using the Wilcoxon rank sum test.

### 2.4 Subjects

Thirty‐two male Sprague–Dawley (SD) rats, aged 7–8 weeks and weighing 180–220 g, were purchased from the experimental center of Beijing Huafukang Co. Ltd. All animal studies were carried out in compliance with the National Academy of Sciences of the National Institutes of Health (NIH) and the guidelines of the Animal Ethical Committee of Jinan University (approval number: IACUC‐20221114‐02). The rats were housed in a climate‐controlled facility under a 12‐h light/dark cycle with free access to food and water.

### 2.5 Cell lines

RSC96 cells were purchased from the Shanghai Cell Institute as a rat Schwann cell line. RAW 264.7 is a mouse macrophage cell line purchased from Guangzhou Yiyou Biotechnology Co., Ltd. Cells were cultured in Dulbecco's modified Eagle's medium (Gibco) with 10% fetal bovine serum (FBS) (Gibco), and 1% penicillin and streptomycin (100 mg/mL) (Gibco) (100 U/mL). Cells were placed in an incubator at 37°C, 5% CO_2_.

### 2.6 Experimental grouping of RSC96 cells and preparation of conditioned medium

In CCK8 experiment, we cultured RSC96 cells with different concentrations of stigmasterol (1–50 μM) and observed that cell viability was highest at concentrations ranging from 1 to 15 μM. Therefore, we used a concentration of 15 μM for stigmasterol in our experiment (Figure [Link], [Link]). RSC96 cells were classified into three distinct groups: control group, LPS group (1 μg/mL LPS), and LPS + stigmasterol group (1 μg/mL LPS + 15 μM stigmasterol). Schwann cells (1.0 × 10^6^) were then seeded onto a 100 mm culture dish and allowed to incubate for 24 h. Following the incubation period, the medium was removed, and 4 mL of LPS‐containing serum‐free medium with a final concentration of 1 μg/mL was added to the culture dish. The mixture was incubated for 24 h, and the supernatant was collected, centrifuged at 1000 *g* for 5 min, and then passed through a 0.22 μm membrane. The collected medium was designated as LPS‐induced Schwann cell‐conditioned medium (L‐CM). Next, the stigmasterol‐treated LPS‐induced Schwann cell‐conditioned medium was collected. Similar to the previous steps, 1.0 × 10^6^ Schwann cells were seeded onto a 100 mm dish and allowed to attach overnight. After removing the medium, 4 mL of serum‐free medium containing both 1 μg/mL of LPS and 15 μM of stigmasterol was added to the dish. After 24 h of treatment, the supernatant was collected, centrifuged at 1000 *g* for 5 min, and then filtered using a 0.22 μm filter. The collected supernatant was then defined as LPS + Stigmasterol‐treated Schwann cell‐conditioned medium (L‐S‐CM).

### 2.7 RAW264.7 cell experiment group

The RAW264.7 cells were divided into seven groups: the control group (unconditioned medium), the L‐CM group (L‐CM conditioned medium), the L‐CM + GW2580 group (L‐CM conditioned medium +5 μM GW2580 [CSF1R inhibitor]), the L‐CM+ SB203850 group (L‐CM conditioned medium +10 μM SB203850 [P38MAPK inhibitor]), the L‐CM + CP‐456773 group (L‐CM conditioned medium +10 μM CP‐456773 [NLRP3 inhibitor]), the L‐S‐CM group (L‐S‐CM conditioned medium), and the Stigmasterol group (Unconditioned Medium +15 μM stigmasterol).

### 2.8 ELISA

The secretion of IL‐34 in the supernatant of RSC96 Schwann cells in the control group, LPS group, and LPS + Stigmasterol group was detected according to the manufacturer's instructions. Blood samples were harvested from the abdominal aortas of the rats and centrifuged at 3000 rpm for 15 min at 4°C. Then, 600 μL of supernatant was immediately transferred to a clean sample tube and stored at −20°C. The interleukin (IL)‐1β, IL‐6, SP, PGE2, CCL2, and TNF‐α levels were measured by ELISA in accordance with the manufacturer's recommendations. Finally, the optical density (OD) was measured using an enzyme marker (Bio Tek Instruments, Inc.).

### 2.9 CCK8 detection cell proliferation assay

After collecting RAW264.7 macrophages, the cell concentration was adjusted to 4–5 × 10^4^ cells/mL, 100 μL of cell suspension was added to each well of a 96‐well plate, and cultured for 24 h. After the cells were observed to adhere to the wall, the corresponding conditioned medium and drugs were added according to the above grouping and then cultured for 24 h. A total quantity of 10 μL of CCK8 solution was added according to the instructions and continued to incubate the 96‐well plate in the incubator for 1 h. Absorbance values were measured at 450 nm on a microplate reader (Thermo Scientific, Waltham, MA, USA).

### 2.10 Cell cycle PI staining

The collected RAW264.7 cells were seeded in a 6‐well culture dish at a density of 1 × 10^6^/well, the cells were treated with the abovementioned conditioned medium and drugs, and the cell suspension was collected. It was first washed with 1 mL of precooled PBS, 2 mL of −20°C precooled 70% ethanol was added to resuspend the cell pellet, and fixed at 4°C for 30 min. After fixation, the cells were washed with PBS again, RNase A (20 μg/mL) was added, and incubated at 37°C for 30 min to completely degrade intracellular RNA. Then, the cell pellet was resuspended with 500 μL PBS, 25 μL of PI staining solution (50 μg/mL) was added, placed on ice and incubated in the dark for 30 min, and then detected with a flow cytometer.

### 2.11 Cell scratch assay

A scratch test was performed to evaluate macrophage migration ability, and RAW264.7 macrophages were seeded into 6‐well plates at a density of 5 × 10^4^ cells/well. When 90% confluence is formed, the monolayer was scraped with a 10 μL pipette tip and washed with PBS to remove detached cells. Cells were incubated with the above conditioned medium for 12 h. Finally, images at 0 h and 12 h were taken with an inverted microscope (magnification, ×40; Carl Zeiss AG, Germany) after washing in PBS. Cell mobility was calculated using ImageJ software and the following equation was used: relative mobility = [width (0 h) − width (12 h)]/width (0 h) × 100%.

### 2.12 Immunofluorescence staining

A slide that had been coated with poly L‐lysine (0.1 mg/mL) was placed in a 6‐well plate. RAW264.7 cells were permeabilized with 0.3% Triton X‐100 for 15 min after being fixed in 4% paraformaldehyde for 30 min at room temperature. The cells were washed three times with PBS, blocked with 1% bovine serum albumin for 1 h, and then incubated with rabbit anti‐Iba‐1 and anti‐CSF1R primary antibodies overnight at 4°C (1:200 dilution). Anti‐fade mounting media containing DAPI was added to the cells and incubated for 10 min at 37°C in the dark. All images were captured with an Olympus fluorescence microscope (BX53) at 400× magnification.

### 2.13 Detection of reactive oxygen species (ROS) in macrophages by DCFH‐DA

The RAW264.7 cell suspension was collected, followed by washing with PBS at 1000 rpm for 5 min. The supernatant was discarded and dilute DCFH‐DA 1:1000 with serum‐free medium to achieve a final concentration of 10 μmol/L. The cells were collected and suspended in the diluted DCFH‐DA at a concentration of 1 million–20 million/mL. The cells were incubated in a 37°C cell culture incubator for 20 min. Subsequently, the cells were washed thrice with serum‐free cell culture medium, ensuring complete removal of the non‐entered DCFH‐DA. Finally, the samples were analyzed by flow cytometry to quantify the intracellular production of ROS.

### 2.14 Neuropathic pain model

A neuropathic pain model of CCI of the sciatic nerve in rats was established based on our previous study.^16^ Initially, a 2% dose of sodium pentobarbital (50 mg/kg) was injected to anesthetize the rats, after which they were fixed to expose the right sciatic nerve. Subsequently, 4.0 sutures were strategically placed under a microscope to ligate the right sciatic nerve at intervals of approximately 1 mm from each other. To prevent infection, the right biceps femoris muscle was then injected with gentamicin (10 mg/mL, i.m.). In contrast, rats in the sham‐operated group only received skin and muscle sutures, and the nerves were not ligated. Increased sensitivity of the injured hind paw to pain after surgery indicated successful modeling of chronic neuralgia.^16^

The 32 rats were randomly assigned into four groups as follows: the sham‐operated group and the CCI group received physiological saline (0.9% at 6 mL/kg) via oral gavage; the CCI + ibuprofen group received ibuprofen (at 63 mg/kg) via oral gavage; and the CCI + stigmasterol group received stigmasterol (at 80 mg/kg)^17^ via oral gavage. It started from the first day after the operation and lasted for 21 days.

### 2.15 Behavioral test

The acetone assay was chosen as the method to assess cold hyperalgesia. Hundred microliters of acetone was dropped onto the plantar surface of the rat's right paw and the total number of times the rat lifted or grasped the right hind paw was counted within a period of 120 s. To evaluate thermal hyperalgesia, the hot plate experiment (YLS‐6B; Jinan Yiyan Technology Development Co., Ltd.) was chosen, whereby the paw thermal withdrawal latency (PTWL) was measured. The hot plate was maintained at a constant temperature of 50°C, and the time taken before the rats raised their right paw was measured. Each ipsilateral paw was tested three times with a rest period of 5 min between each heat treatment, and the mean value was calculated. The right paw hyperalgesia was measured 1 day before surgery (Day 0) and was considered the preoperative value. Furthermore, hyperalgesia of the right paw was assessed on postoperative Days 1, 4, 7, 14, and 21 after surgery.

### 2.16 Hematoxylin–eosin staining

The rats' DRG, livers, kidneys, and right sciatic nerves were fixed for 24 h in 4% paraformaldehyde and subsequently embedded in paraffin and sectioned at a thickness of 3 μm. The sections were dewaxed in xylene and rehydrated using a series of ethanol concentrations (100%, 90%, 80%, and 70%). Subsequently, the sections were washed in PBS for 5 min. Photographs of the stained sections were evaluated by two blinded pathologists to determine tissue damage. An Olympus fluorescence microscope (BX53) was used to examine the pathological sections.

### 2.17 Immunohistochemical assessment

The DRG of rats were fixed in 4% paraformaldehyde for 24 h. Following fixation, the tissues were subjected to two washes for dehydration and subsequently embedded in paraffin, prior to being sliced into 3‐μm‐thick sections. Following blocking of nonspecific protein interactions with 3% bovine serum albumin solution for 30 min, antigen retrieval was performed using a sodium citrate antigen repair solution (1:1000 dilution, pH = 6). The sections were then incubated with primary antibodies overnight at 4°C, before being incubated with the corresponding secondary antibodies. Following this, the sections were washed with 0.1 M PB and treated with DAB. The sections were then cleared, dehydrated, washed with distilled water, and xylene sealed. Subsequently, images were captured using an Olympus fluorescence microscope (BX53) with an approximate magnification of 400×. Cells that exhibited yellowish‐brown color were considered positive. Mean optical density was analyzed using Image‐Pro Plus 6.0.

### 2.18 Western blot analysis

Total protein was extracted from RSC96 and RAW2647 in RIPA lysis buffer containing 1 mM phenylmethylsulfonyl fluoride (PMSF) and centrifuged at 10,000 *g* for 10 min. The DRG were extracted from rats in RIPA lysis buffer containing 1 mM PMSF and centrifuged at 12,000 *g* for 15 min. Western blotting was used to measure the expression levels of IL‐34, CSF1R, NFkB, pNFkB, P38 MAPK, pP38MAPK, NLRP3, ASC, IL‐1β, IL‐18, Iba‐1, and Caspase 1. Images were captured utilizing the ChemiDoc MP imaging system (Bio‐Rad). Each experiment was run at least three times.

### 2.19 Quantitative real‐time PCR (qRT–PCR)

Total RNA was extracted via RNAiso Plus, and resultant cDNA was synthesized using a qRT–PCR kit following the manufacturer's instructions. The amplification conditions were 95°C for 30 s, then 40 cycles of 95°C for 5 s and 60°C for 34 s, 95°C for 15 s, 60°C for 60 s, and 95°C for 15 s on the BIO‐RAD CFX96 Real‐Time PCR System (BIO‐RAD, USA). The relative expression of mRNA was determined using the $2^{-\Delta \Delta C_{t}}$ method after normalization to the expression of β‐actin. All the primers used are shown in Table 1.

**TABLE 1:** Primer sequences.

Genes	Forward primer (5′→3′)	Reverse primer (5′→3′)
ASC	AGACATCGGGAGGATTTAC	GAGCACCACACTCAAGG
β‐Actin	CCTAGACTTCGAGCAAGAGA	GGAAGGAAGGCTGGAAGA
Caspase1	TGAAAGACAAGCCCAAGGT	GAAGAGCAGAAAGCAATAAAA
IL‐1β	AGGAGAGACAAGCAACGACA	CTTTTCCATCTTCTTCTTTGGGTAT
IL‐6	AGTTGCCTTCTTGGGACTGATGT	GGTCTGTTGTGGGTGGTATCCTC
IL‐18	CTGGCTGTGACCCTATCTG	AAGCATCATCTTCCTTTTGG
TNF‐α	GCGTGTTCATCCGTTCTCTACC	TACTTCAGCGTCTCGTGTGTTTCT
NLRP3	CTGTCTCACATCTGCGTGTT	GTCTCCCAAGGCATTTTCT

### 2.20 Molecular docking

The 2D structure of the ligand (molecule) was retrieved from PubChem (https://pubchem.ncbi.nlm.nih.gov/). The 3D structures of the receptor (protein), namely IDO1(3JS2) were downloaded from the RCSB PDB database (http://rcsb.org/). The molecular docking was performed using the AutoDock Tools 1.5.6 and AutoDock Vina plug‐in. Binding energy was used to evaluate the results of molecular docking, and PyMol was used to visualize the molecular docking results.

### 2.21 Statistical analyses

Every experiment were performed in triplicate, and the results were statistically analyzed using GraphPad Prism 9.0 (GraphPad Software, CA) and R 4.0.5 software. The mean ± (SEM) was presented to summarize the data. One‐way analysis of variance (ANOVA) and two‐way ANOVA, followed by post hoc (Bonferroni) tests, were performed for multiple group comparisons when the data exhibited normal distribution with uniform variance. Nonparametric tests were used if the data did not conform to a normal distribution. The significance was set at *p* < 0.05 to identify statistically significant differences.

## 3 RESULTS

### 3.1 Increased proportion of macrophages in DRG increases after peripheral nerve injury (Figure 1)

**FIGURE 1:**
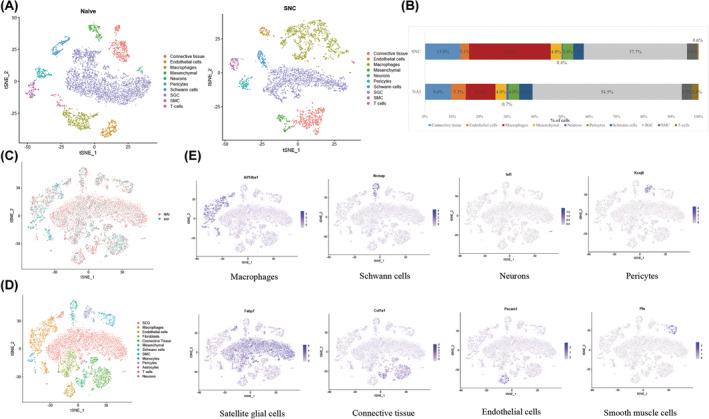
Naive and SNC mouse DRG cell annotation results (A) Annotated t‐SNE plot of DRG cells isolated from Naive mice and SNC mice. (B). Proportion of each cell type in Naive mice and SNC mice (C) After merging Naive and SNC DRG data annotated results (grouped by Naive and SNC). (D) Annotation results (shown by cell type) after merging Naive and SNC DRG data. (E) Macrophages (Aif1/Iba1), Schwann cells (Ncmap), pericytes (Kcnj8), neurons (Isl1), SGCs (Fabp7), endothelial cells (Pecam), connective tissue (Col1a1), and smooth muscle t‐SNE plot of cells (Pln). Relative expression levels are shown as purple gradients on the left.

Single‐cell RNA sequencing analysis was conducted on the L4 dorsal root ganglia (DRGs) of mice with sciatic nerve injury (SNC), utilizing the contralateral uninjured L4 DRG as a control. This study found that in Naive mice, connective tissue cells, endothelial cells, macrophages, mesenchymal cells, neurons, pericytes, Schwann cells, satellite glial cells (SGCs), smooth muscle cells (SMCs), and T cells accounted for 9.6%, 5.3%, 10.9%, 4.0%, 0.7%, 4.0%, 4.8%, 54.5%, 3.7%, and 2.4%, respectively. In contrast, the proportions of these cells in the DRG of SNC mice were 13.0%, 3.1%, 29.8%, 4.0%, 0.4%, 3.8%, 3.9%, 37.7%, 3.6%, and 0.6%, respectively (Figure 1A,B). Notably, the proportion of macrophages increased significantly in the DRG of mice in the SNC group compared with those in the Naive group. The distribution of two sets of cell annotations after the merger of Naive and SNC is illustrated in Figure 1C, while Figure 1D displays the result of cell annotation after the merger of Naive and SNC. To ensure the accuracy of labeling, markers for various cell types were examined (Figure 1E), with the results indicating that the proportion of macrophages in the DRG was significantly higher after peripheral nerve injury as compared to the uninjured DRG.

### 3.2 Schwann cells in DRG secrete IL‐34 and act on macrophages after peripheral nerve injury

**FIGURE 2:**
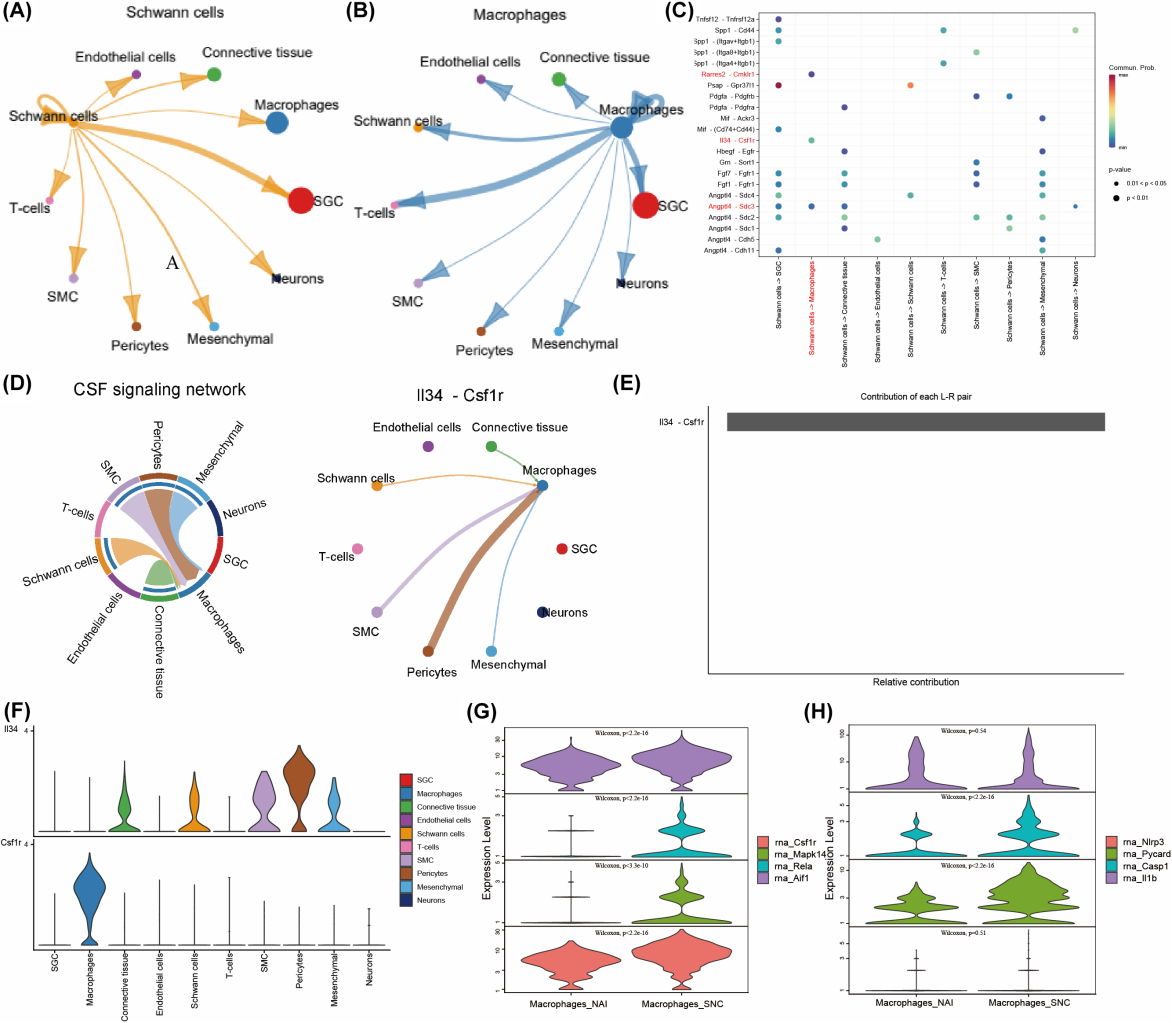
Analysis of cell communication among DRG cells and expression of CSF signaling pathway. (A) Schwann cell interaction diagram. (B) Macrophage bar interaction diagram. (C) Ligand–receptor bubble diagram (D) CSF signaling pathway chord diagram (E) IL‐34/SF1R ligand contribution (F) Violin plot of expression levels of IL‐34 and CSF1R in different cell types. (G) Violin plot of expression levels of CSF1R (Csf1r), P38 Mapk (Mpak14), NF‐κB (Rela), and Iba‐1 (Aif1) (H). Violin plot of ASC (Pycard), Caspase‐1 (Casp1), NLRP3 (Nlrp3), and IL‐1β (Il1b) protein expression.

In order to comprehend the interaction between different cell types in DRG after sciatic nerve injury, the “CellChat” R package was employed to analyze the cell communication network in the DRG of SNC mice. Figure 2A,B showed the interaction of Schwann cells, macrophages, and other cells. Macrophages and Schwann cells play an important role in peripheral sensitization, so this study will focus on the ligand–receptor interaction between Schwann cells and macrophages. The results showed that IL‐34, Angptl4, and Rarres2 secreted by Schwann cells acted on CSF1R, Sdc3, and Cmklr1 of macrophages, respectively (Figure 2C). The CSF signaling pathway is related to the proliferation, activation, and migration of macrophages, so this study focused on the CSF signaling pathway.^8^ Figure 2D showed that IL‐34 secreted by Schwann cells acted on CSF1R of macrophages to activate CSF signaling pathway. Ligand contribution of CSF signaling suggests that IL‐34 is the major ligand for CSF1R. CSF1, another ligand for CSF1R, did not contribute (Figure 2E). It shows that Schwann cells act on the CSF1R receptor of macrophages by secreting IL‐34, rather than colony‐stimulating factor 1 (CSF1). Figure 2F shows the expression levels of IL‐34 and CSF1R in each cell type. CSF1R is only expressed in macrophages, while IL‐34 is expressed not only in Schwann cells but also in pericytes, smooth muscle cells, connective tissue, and mesenchymal cells. This implies that following sciatic nerve injury, Schwann cells in DRG activate the CSF signaling pathway of macrophages mainly through IL‐34 but not through CSF1.

### 3.3 Difference analysis of IL34/CSF1R signaling pathway

We detected the expression of CSF1R and its downstream P38MAPK and NFκB, respectively, to understand the activation of CSF1R downstream signaling. The results showed that the expression levels of macrophage CSF1R, P38 Mapk (Mpak14), and NFκB (Rela) were increased in the DRG of SNC mice. The expression level of macrophage activation marker Iba‐1 (Aif1) was also increased (Figure 2G, *p* < 0.05). Meanwhile, the expression levels of Caspase1 and Pycard (ASC) in NLRP3 inflammasome were increased (Figure 2H, *p* < 0.05). These results indicate that Schwann cells may affect the proliferation of macrophages and the activation of NLRP3 inflammasome through the IL‐34/CSF1R/P38MAPK/NFκB signaling pathway after peripheral sciatic nerve injury.

### 3.4 Stigmasterol can reduce IL‐34 secreted by LPS‐induced Schwann cells

**FIGURE 3:**
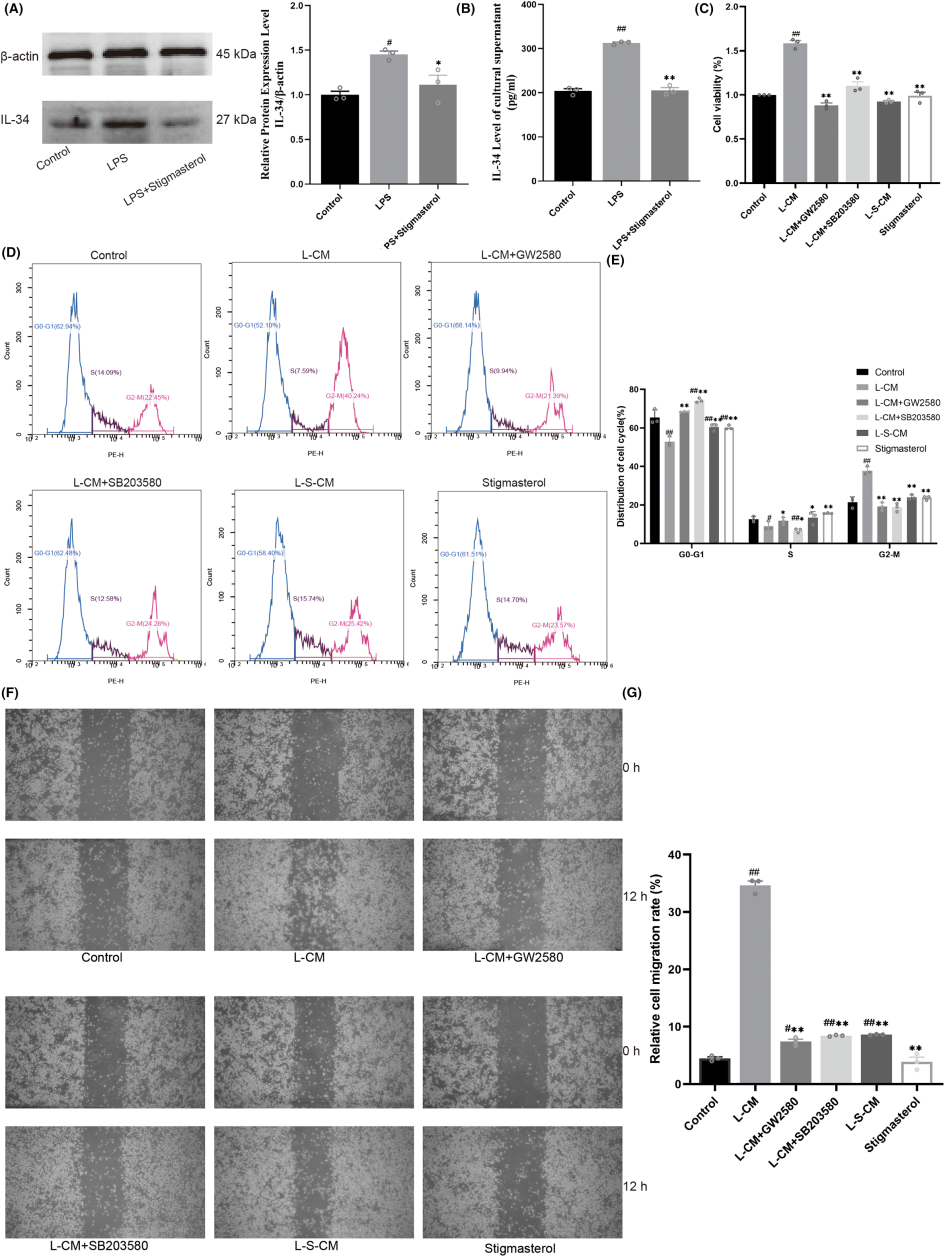
The relationship between the secretion level of IL‐34 of RSC96 Schwann cells and the proliferation and migration of RAW264.7 macrophages. (A) Western blot results of IL‐34 in RSC96 Schwann cells. (B) ELISA detection of IL‐34 concentration in the supernatant of RSC96 Schwann cells. (C) CCK8 detects the proliferation activity of RAW264.7 macrophages. (D, E) PI staining cell cycle results of RAW264.7 macrophages. (F, G) Results of cell scratch experiment of RAW264.7 macrophages (*n* = 3, ^#^ *p* < 0.05, ^##^ *p* < 0.01 vs. Control group, *p < 0.05, **p < 0.01 vs. L‐CM group).

First, the protein levels of IL‐34 in RSC96 cells of each group were detected by western Blot. The results showed that the IL‐34 protein level of LPS‐induced RSC96 cells was significantly higher than that of the control group. The IL‐34 protein level of LPS‐induced RSC96 cells treated with stigmasterol was significantly lower than that of LPS group (Figure 3A, *p* < 0.05). Next, the concentration of secreted IL‐34 in the supernatant of RSC96 cells from each respective group was determined using the ELISA. It was found that the concentration of IL‐34 in the supernatant of LPS‐induced RSC96 cells was significantly higher than that of the control group. The concentration of IL‐34 in the supernatant of LPS‐induced RSC96 cells treated with stigmasterol was significantly reduced (Figure 3B, *p* < 0.05). It shows that stigmasterol can inhibit the secretion of IL‐34 in LPS‐treated Schwann cells.

### 3.5 Stigmasterol‐treated LPS‐induced Schwann cell‐conditioned medium reduces macrophage proliferation and migration

To understand whether the secretion of IL‐34 by RSC96 cells leads to the proliferation and migration of RAW264.7 cells, we performed CCK8, PI cell cycle staining, and Cell scratch assay. The results of CCK8 showed that compared with the control group, RAW 264.7 cells proliferated under L‐CM stimulation; after L‐CM stimulation, treatment with GW2580 (CSF1R inhibitor) and SB203580 (P38MAPK inhibitor) inhibited the proliferation of RAW 264.7 cells; L‐S‐CM treatment did not see significant proliferation of RAW 264.7 cells (Figure 3C, *p* < 0.05). The results of PI cell cycle staining showed that compared with the control group, RAW264.7 cells treated with L‐CM proliferated actively in the G2‐M phase (Figure 3D,E, *p* < 0.05). Compared with L‐CM group, GW2580 and SB203580 could reduce the proliferation process of RAW 264.7 cells in G2‐M phase induced by L‐CM. However, L‐S‐CM did not significantly induce the proliferation of RAW 264.7 cells in the G2‐M phase. It indicated that L‐CM treatment would increase the proportion of macrophages in the proliferative phase, but the proliferation of macrophages treated with L‐S‐CM was not obvious.

The results of the Cell scratch assay showed that, compared with the control group, the signs of cell migration after L‐CM treatment were significantly increased. Compared with the L‐CM group, treatment with GW2580 and SB203580 after L‐CM stimulation significantly inhibited the migration signs of RAW264.7 cells (Figure 3F,G, *p* < 0.05). There was no obvious sign of cell migration in RAW 264.7 after L‐S‐CM treatment. This suggested that L‐CM treatment could promote the migration ability of macrophages, while L‐S‐CM could not obviously promote the migration ability of macrophages.

### 3.6 Immunofluorescence co‐expression analysis of Iba‐1 and CSF1R in macrophages

**FIGURE 4:**
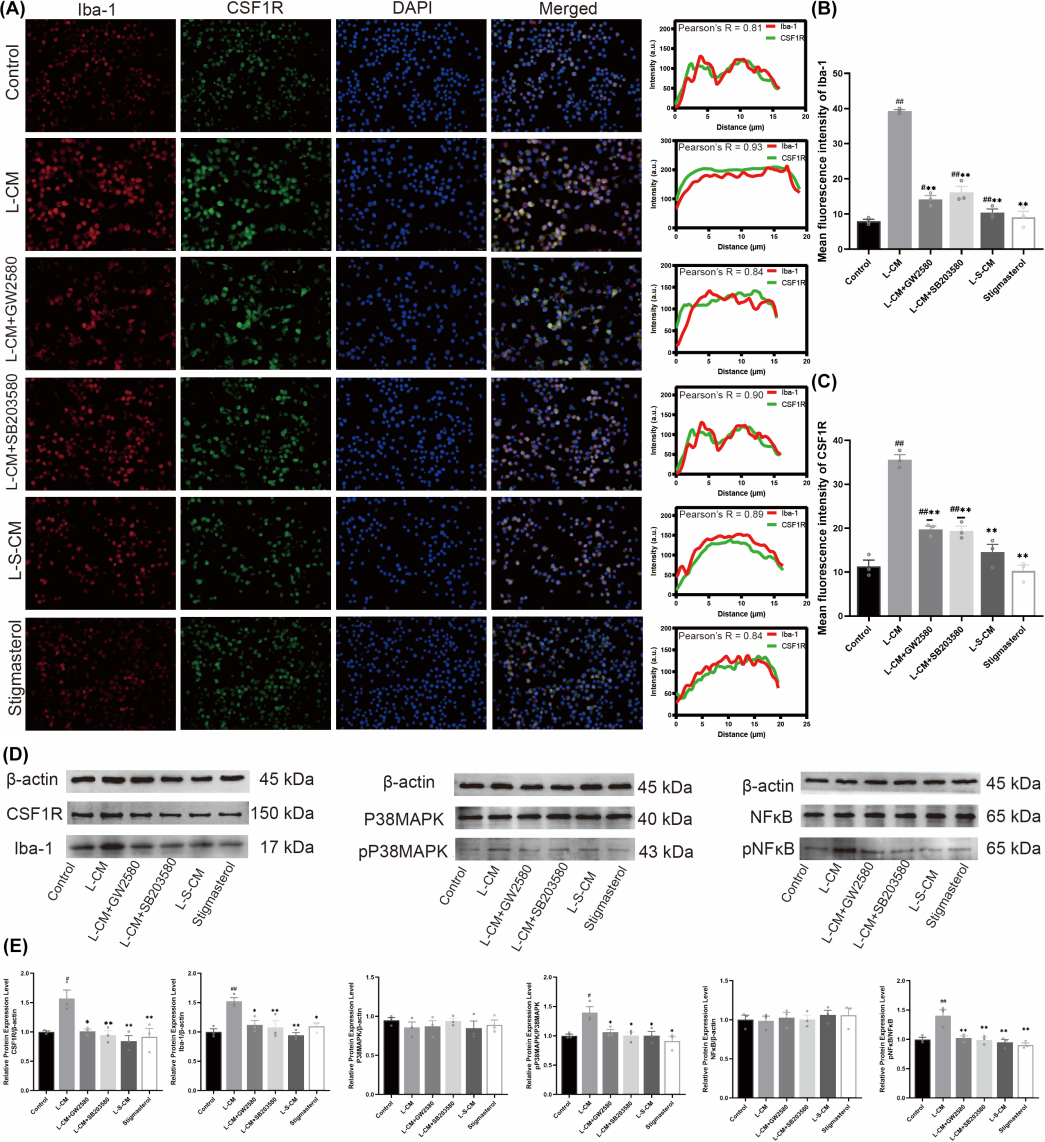
CSF1R and Iba‐1 immunofluorescence colocalization analysis and western blot results of CSF1R/P38MAPK/NFκB signaling pathway. (A) Immunofluorescence colocalization results of CSF1R and Iba‐1. (B, C) Immunofluorescence intensity analysis of CSF1R and Iba‐1. (D, E) Western blot results for CSF1R, Iba‐1, P38MAPK, pP38MAPK, NFκB, and pNFκB (*n* = 3, ^#^ *p* < 0.05, ^##^ *p* < 0.01 vs. Control group, *p < 0.05, **p < 0.01 vs. L‐CM group).

Iba‐1 is a specific marker of macrophage activation, and CSF1R plays an important role in differentiation and survival of macrophages.^18^ We evaluated the colocalization of Iba‐1 and CSF1R expression, and the correlation analysis of each group showed that the Pearson correlation coefficients was >0.6, indicating that Iba‐1 and CSF1R were co‐expressed in RAW 264.7 macrophages (Figure 4A–C). Immunofluorescence image results showed that L‐CM increased the fluorescence intensity of Iba‐1 and CSF1R in RAW 264.7 cells, while administration of GW2580 and SB203580 both weakened the fluorescence intensity of Iba‐1 and CSF1R. Immunofluorescence for L‐S‐CM treatment was not apparent.

### 3.7 Stigmasterol‐treated LPS‐induced Schwann cell‐conditioned medium inhibits activation of CSF1R/P38MAPK/NF‐κB pathway in macrophages

We examined the protein expression of CSF1R/P38MAPK/NFκB in RAW264.7 macrophages by western blot. The results showed that compared with the control group, L‐CM treatment significantly increased the expression levels of CSF1R and Iba‐1 (Figure 4D,E, *p* < 0.05). There was no significant difference in the expression levels of P38MAPK and NFκB among the groups. Compared with L‐CM group, GW2580 and SB203580 downregulated the expressions of CSF1R, Iba‐1, pP38 MAPK, and pNFκB (Figure 4D,E, *p* < 0.05). L‐S‐CM treatment did not significantly increase the expression levels of the above proteins. This revealed that stigmasterol‐treated LPS‐induced Schwann cell‐conditioned medium could inhibit the activation of CSF/P38MAPK/NFKB signaling pathway, thereby reducing the activation of RAW264.7 macrophages.

### 3.8 Stigmasterol‐treated LPS‐induced Schwann cell‐conditioned medium inhibits NLRP3 inflammasome in macrophages

**FIGURE 5:**
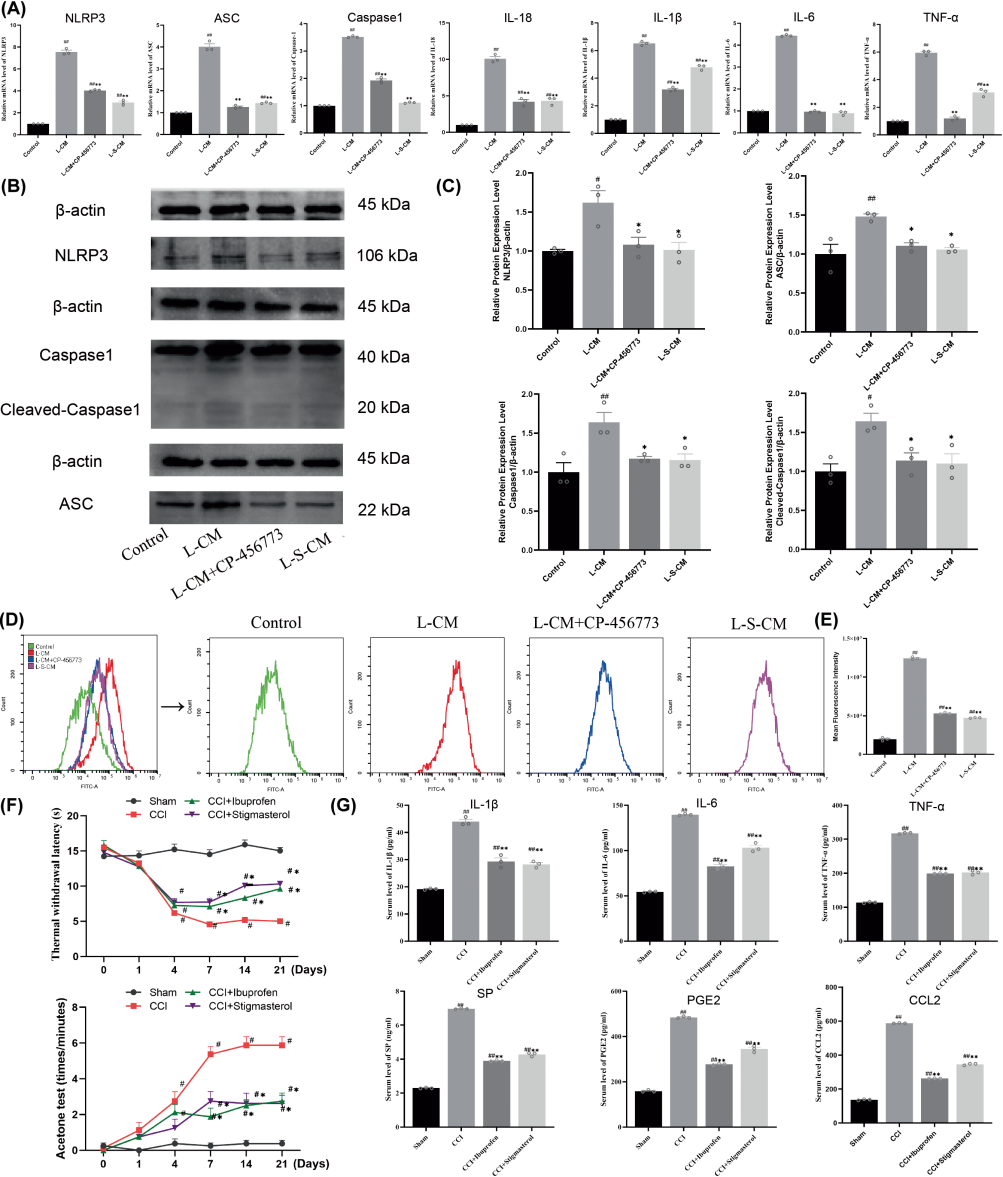
NLRP3 inflammasome expression and ROS level in RAW264.7 macrophages, CCI rat behavior, and serum ELISA results. (A) The mRNA expression of NLRP3 inflammasome in RAW264.7 macrophages. (B, C) Western blot detection of the expression of NLRP3 inflammasome in RAW264.7. (D, E) Flow cytometry detection of ROS in RAW264.7 macrophages (*n* = 3, #*p* < 0.05, ##*p* < 0.01 vs. Control group, *p < 0.05, ** *p* < 0.01 vs. L‐CM group). (F) Hot plate test and acetone test results of CCI rats. (G) Concentrations of IL‐1β, IL‐6, TNF‐α, SP, PGE2, and CCL2 in rat serum detected by ELISA (*n* = 3, ^#^ *p* < 0.05, ^##^ *p* < 0.01 vs. Sham group, *p < 0.05, **p < 0.01 vs. CCI group).

We detected the expression levels of NLRP3 inflammasome in each group by western blot and RT‐PCR. The results showed that the mRNA expression levels of NLRP3, ASC, Caspase 1, IL‐1β, IL‐6, TNF‐α, and IL‐18 in the L‐CM group were significantly higher than control group. CP‐456773 (NLRP3 inhibitor) treatment decreased the expression levels of these mRNAs (Figure 5A, *p* < 0.05). However, L‐S‐CM treatment did not significantly increase the mRNA expressions of NLRP3, ASC, Caspase1, IL‐1β, IL‐6, TNF‐α, and IL‐18 (Figure 5A, *p* < 0.05). Western blot showed that, compared with the control group, the protein expression levels of NLRP3, ASC, and Caspase1 in the L‐CM group were significantly increased (Figure 5B,C, *p* < 0.05). Compared with the L‐CM group, the CP‐456773 treatment reversed this trend. The protein expression levels of NLRP3, ASC, Caspase1, and Cleaved‐Caspase1 were not significantly increased after L‐S‐CM treatment. This suggests that stigmasterol‐treated LPS‐induced Schwann cell‐conditioned medium can reduce the activation of NLRP3 inflammasome in RAW264.7 macrophages.

### 3.9 Stigmasterol‐treated LPS‐induced Schwann cell‐conditioned medium can reduce the level of ROS in macrophages

Flow cytometry was used to detect the level of ROS in RAW264.7 macrophages treated with L‐CM. The results showed that the L‐CM group induced a significant upregulation of ROS levels in RAW 264.7 macrophages compared with the control group (Figure 5D,E, *p* < 0.05). Compared with the L‐CM group, CP‐456773 significantly downregulated the level of ROS, while L‐S‐CM treatment significantly reduced ROS levels. The results suggested that stigmasterol‐treated LPS‐induced Schwann cell‐conditioned medium could reduce the level of ROS in RAW264.7 macrophages.

### 3.10 Stigmasterol attenuates thermal and cold hyperalgesia in CCI rats

The day before surgery, thermal and cold hyperalgesia were measured in all rats, all rats were equally sensitive to hyperalgesia. Rats developed severe thermal and cold hyperalgesia on day 4 after CCI surgery. Stigmasterol relieved thermal and cold hyperalgesia from day 7 onward. Stigmasterol increased pain threshold but did not restore normal pain sensitivity (Figure 5F, *p* < 0.05).

### 3.11 Stigmasterol reduces serum inflammatory factor levels in CCI rats

We measured the serum IL‐1β, IL‐6, TNF‐α, CCL2, PGE2, and SP concentrations of rats in each group by ELISA. The results showed that the serum levels of IL‐1β, IL‐6, TNF‐α, CCL2, PGE2, and SP in the CCI group were higher than those in the sham group. Compared with the CCI group, ibuprofen and stigmasterol decreased the levels of these pro‐inflammatory factors in serum (Figure 5G, *p* < 0.05). This finding suggests that stigmasterol can reduce the levels of serum inflammatory factors in CCI rats.

### 3.12 Stigmasterol reduces inflammatory cell infiltration and decreases CSF1R, Iba‐1, and NLRP3 expression in DRG of CCI rats

**FIGURE 6:**
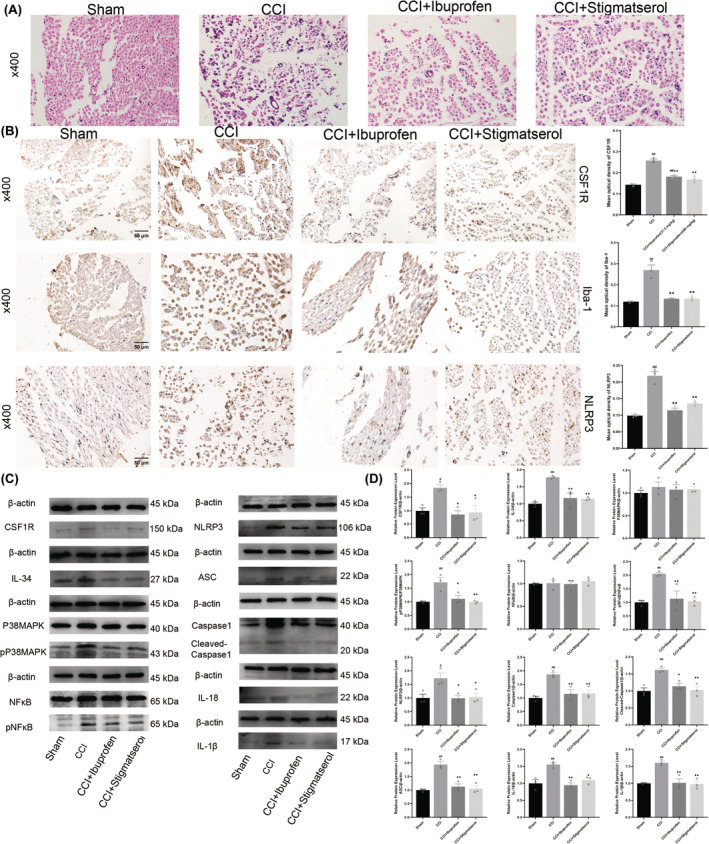
H&E, immunohistochemistry, and western blot results of rat DRG. (A) H&E results of rat DRG. (B) Immunohistochemical results of CSF1R, NLRP3, and Iba‐1 of rat DRG. (C, D) The expression of CSF1R, IL‐34, P38MAPK, pP38MAPK, NFκB, pNFκB, NLRP3, ASC, Caspase‐1, Cleaved‐Caspase1, IL‐1β, and IL‐18 in rats detected by western blot. Scale bar = 50 μm (*n* = 3, ^##^ *p* < 0.01 vs. Sham group, *p < 0.05, ** *p* < 0.01 vs. CCI group).

We observed a disorderly structure of the sciatic nerve in rats who underwent CCI. In addition, the results revealed significant infiltration of inflammatory cells within the sciatic nerve tissue. These findings demonstrate the successful induction of a neuropathic pain model in rats using the CCI surgery (Figure S1A). We also found that there were many kinds of inflammation cell infiltration in the DRG after CCI. The level of inflammatory cell infiltration in the stigmasterol group and the ibuprofen group was significantly lower than that in the CCI group (Figure 6A). The structures of the liver and kidney in each group were normal, indicating that drug administration and CCI surgery had no adverse effects on the liver and kidney of the rats (Figure S1B,C). We used immunohistochemistry to observe the expression levels of CSF1R, Iba‐1, and NLRP3. We found that the expression levels of CSF1R, Iba‐1, and NLRP3 were significantly increased in the DRG of CCI rats. Stigmasterol and ibuprofen reversed these trends (Figure 6B).

### 3.13 Stigmasterol reduces activation of CSF1R/P38MAPK/NFκB pathway and NLRP3 inflammasome in DRG of CCI rats

Western blot shows that compared with the Sham group, the expression levels of CSF1R, IL‐34, Pp38MAPK, pNFκB NLRP3, ASC, Caspase‐1, IL‐1β, and IL‐18 in the DRG of CCI rats were significantly increased (Figure 6C,D, *p* < 0.05). The levels of P38MAPK and NF‐κB were not significantly different among the groups. Compared with the CCI group, the expression levels of CSF1R, IL‐34, NLRP3, ASC, Caspase‐1, IL‐1β, IL‐18, pP38 MAPK, and pNFκB were downregulated after administration of ibuprofen and stigmasterol (Figure 6C,D, *p* < 0.05). It shows that stigmasterol can reduce the activation of CSF1R/P38MAPK/NFKB pathway and the activation of NLRP3 inflammasome in DRG of CCI rats.

## 4 DISCUSSION

In this study, we provided the following evidence: (1) Cellchat analysis revealed that Schwann cells secrete IL‐34 to stimulate the CSF1R receptor of macrophages. (2) mRNA expression levels of the CSF1/P38MAPK/NFκB signaling pathway and NLRP3 inflammasome in phagocytes were increased in DRG macrophages following peripheral nerve injury. (3) Macrophage exposure to LPS‐induced Schwann cell‐conditioned medium (L‐CM) resulted in the promotion of proliferation and migration, while treatment with stigmasterol of LPS‐induced Schwann cell‐conditioned medium (L‐S‐CM) showed an inhibitory effect on this phenomenon. (4) L‐S‐CM inhibited the activation of the CSF1R/P38MAPK/NFκB pathway in macrophages exposed to L‐CM; (5) L‐S‐CM reduced the activation of NLRP3 inflammasome and ROS production in macrophages exposed to L‐CM; and (6) stigmasterol was found to inhibit the activation of the CSF1R/P38MAPK/NFκB signaling pathway and NLRP3 inflammasome in DRG of CCI rats.

A cascade of Schwann cells and macrophages plays a crucial role in NP.^19^ Following peripheral nerve injury, Schwann cells produce and release nerve mediators, such as cytokines, chemokines, growth factors, and biologically active small molecules, to recruit immune cells such as macrophages to the site of injury.^20^ Schwann cells and macrophages in DRG co‐secrete cytokines (TNF‐α, IL‐1β, and IL‐6), chemokines (CCL2 and COX2), growth factors (NGF and BDNF), and ATP, mediating peripheral sensitization, increasing neuronal synaptic excitability, and reducing pain threshold.^21^ Through Cellchat analysis of mice's DRG, an increased proportion of macrophages in sciatic nerve injury compared with the uninjured site was found. Schwann cells in DRG secrete IL‐34, acting on the CSF1R receptor of macrophages after PNI. The mRNA expression of CSF pathways (CSF1R, P38MAPK, and NFκB) and NLRP3 inflammasome were elevated in DRG macrophages with sciatic nerve injury.

CSF1R plays a crucial role in the proliferation, differentiation, and survival of macrophages.^18^ Research indicates that the binding of IL‐34 to CSF‐1R can activate the P38MAPK and NFκB signaling pathways in macrophages.^8^ P38MAPK and NFκB are important signaling pathways that promote macrophage proliferation and survival, as well as induce macrophages to release inflammatory cytokines (IL‐1β, TNF‐α, and IL‐6).^22^ We utilized ELISA and western blotting found that RSC96 cells secreted a large quantity of IL‐34 after LPS treatment, and stigmasterol reduced the secretion of IL‐34. Next, we collected L‐CM and L‐S‐CM for further experiments. Through CCK8, PI cell cycle staining, and Cell scratch assay, we found that L‐CM treatment promoted the proliferation and migration of RAW 264.7 cells, while L‐S‐CM treatment did not exhibit obvious signs of proliferation and migration of RAW 264.7 cells. Western blotting experiments revealed that the protein levels of CSF1R, p38MAPK, and pNFκB were increased in macrophages cultured in L‐CM. However, the levels of these proteins were lower in the L‐S‐CM group as compared to the L‐CM group, indicating that stigmasterol reduced the activation of the CSF1R/P38MAPK/NFKB pathway in subsequent macrophages by regulating the secretion of IL‐34 from Schwann cells. The molecular docking results indicate that stigmasterol does not form hydrogen bonds tightly with CSF1R (Figure S2). Therefore, stigmasterol may potentially decrease the secretion and expression of IL‐34 in Schwann cells, thereby reducing its binding to macrophage CSF1R and influencing the proliferation and migration of macrophages. However, stigmasterol may not directly act on the macrophage CSF1R receptors to affect their homeostasis.

The activation of NLRP3 inflammasome in macrophages has been known to mediate neuroinflammation that plays a crucial role in maintaining NP.^23^ The NLRP3 inflammasome complex comprises NLRP3, ASC, and pro‐caspase 1, which generate active Caspase 1 through their interactions.^24^ The activated Caspase 1 undergoes a cleavage process and converts pro‐IL‐1β and pro‐IL‐18 into biologically active IL‐1β and IL‐18.^25^ In our study, we observed an increased expression of NLRP3, ASC, Caspase‐1, IL‐1β, and IL‐18 in macrophages cultured in L‐CM. However, the macrophages treated with L‐S‐CM showed a decrease in the expression levels of NLRP3, ASC, Caspase‐1, IL‐1β, and IL‐18. These findings suggest that stigmasterol inhibits the activation of NLRP3 inflammasome in macrophages by reducing the secretion of IL‐34 by Schwann cells. Additionally, energy metabolism and NLRP3 inflammasome activation in macrophages result in the production of ROS, which are considered to be a crucial factor in inducing NP.^26^ Through flow cytometry analysis, we found a significant increase in the level of ROS in macrophages treated with L‐CM compared to the control group. Conversely, the level of ROS in macrophages cultured with L‐S‐CM was lower than that of macrophages in the L‐CM group. These findings demonstrate that L‐S‐CM can effectively inhibit the activation of NLRP3 and the subsequent production of ROS in macrophages.

In vitro experiments demonstrated that stigmasterol decreases the secretion of IL‐34 in RSC96 Schwann cells, thereby inhibiting the CSF1R/P38MAPK/NFκB signaling pathway and NLRP3 inflammasome in RAW264.7 macrophages. In vivo validation was performed using CCI model rats. Behavioral experiments indicated that stigmasterol has the ability to reduce thermal and cold hyperalgesia in CCI rats. ELISA tests revealed a reduction in levels of pro‐inflammatory factors (IL‐1β, IL‐6, and TNF‐α)^27^ and pain‐related mediators (SP, PGE2, and CCL2)^28^, ^29^ in the serum of CCI rats treated with stigmasterol. This data suggests that stigmasterol reduces chronic pain through its anti‐inflammatory properties. Immunohistochemistry and western blot tests demonstrated that stigmasterol decreases the expression of IL‐34/CSF1R/P38MAPK signaling and the NLRP3 inflammasome in DRG of CCI rats.

Our findings suggest that stigmasterol could hinder the Schwann cells and macrophages cascade via the IL‐34/CSF1R axis, leading to a decrease in neuropathic pain in CCI rats and ultimately curbing neuroinflammation and reducing peripheral sensitization of DRG.

## 5 CONCLUSION

Our study reveals that stigmasterol can alleviate neuropathic pain by suppressing the IL‐34/CSF1R axis in Schwann cells and macrophages.

## AUTHOR CONTRIBUTIONS

Waimei Si, Xin Li, Di Zhang, and Guoping Zhao played critical roles in conceptualizing the experiment, data analysis, and carrying out the experimental procedures. Furthermore, Zhenni Chen, Bei Jing, YaChun Zheng, and Shiquian Chang provided assistance with English language editing. Correspondingly, Di Zhang and Guoping Zhao are credited as the corresponding authors. All authors participated in the review process and ultimately approved the final article.

## FUNDING INFORMATION

This work was supported by the National Natural Science Foundation of China, Grant/Award Numbers: 82105047 and 82274294; the China Postdoctoral Science Foundation, Grant/Award Number: 2023TQ0135; and the Postdoctoral Fellowship Program of CPSF, Grant/Award Number: GZC20230979.

## CONFLICT OF INTEREST STATEMENT

There are no conflicts of interest.

## Supporting information


Figure S1.

Figure S2.

Figure S3.

## Data Availability

The data used to support the findings of this study are available from the corresponding author upon request.
