# Supplementary figures and images for: Stigmasterol alleviates neuropathic pain by reducing Schwann cell‐macrophage cascade in DRG by modulating IL‐34/CSF1R
Source: CNS Neurosci Ther. 2024 Apr 4;30(4):e14657. doi: 10.1111/cns.14657 (PMC10993342; doi:10.1111/cns.14657)

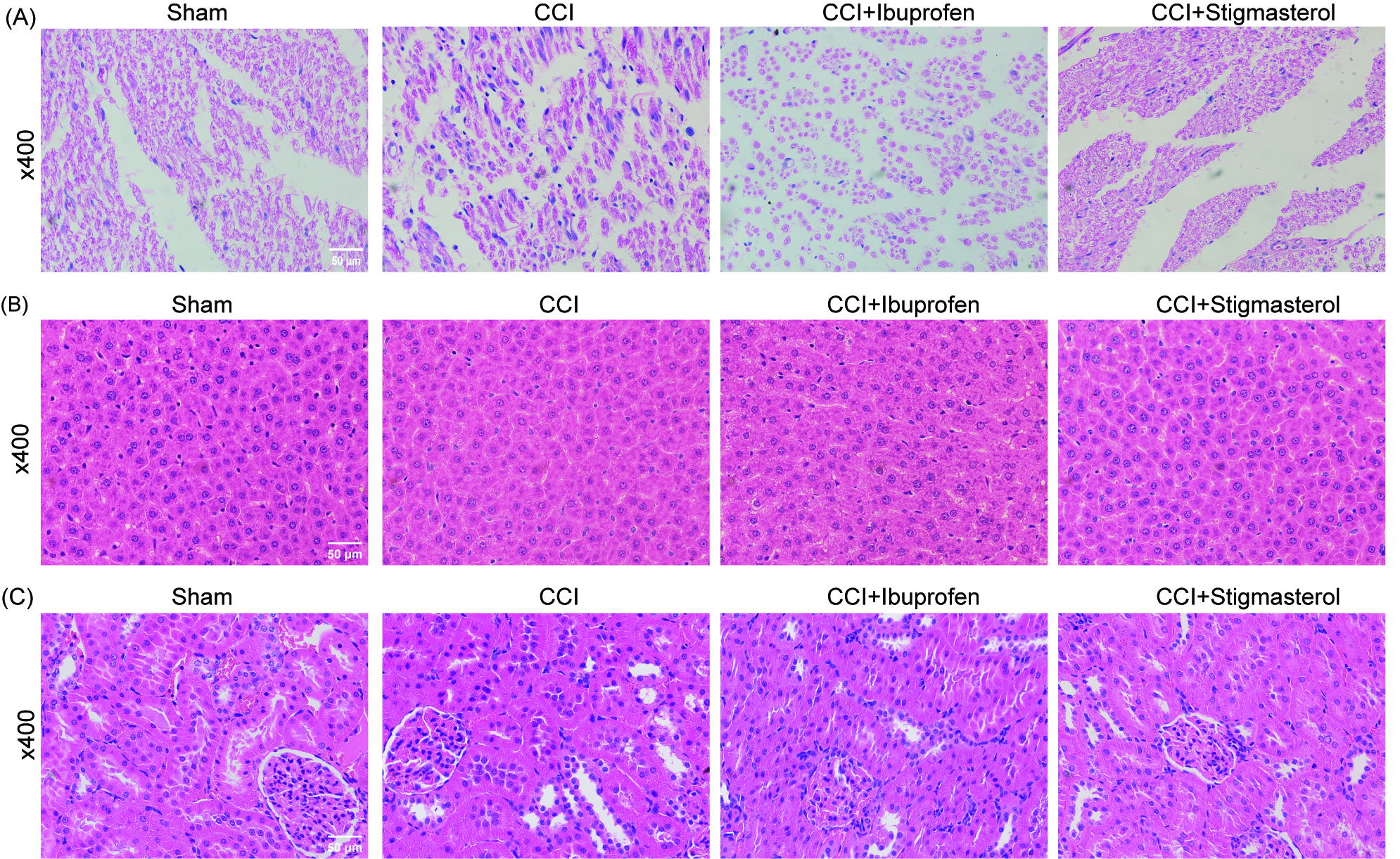

Supplement: Supplementary file 1 — Figure S1. [file CNS-30-e14657-s002.tif]

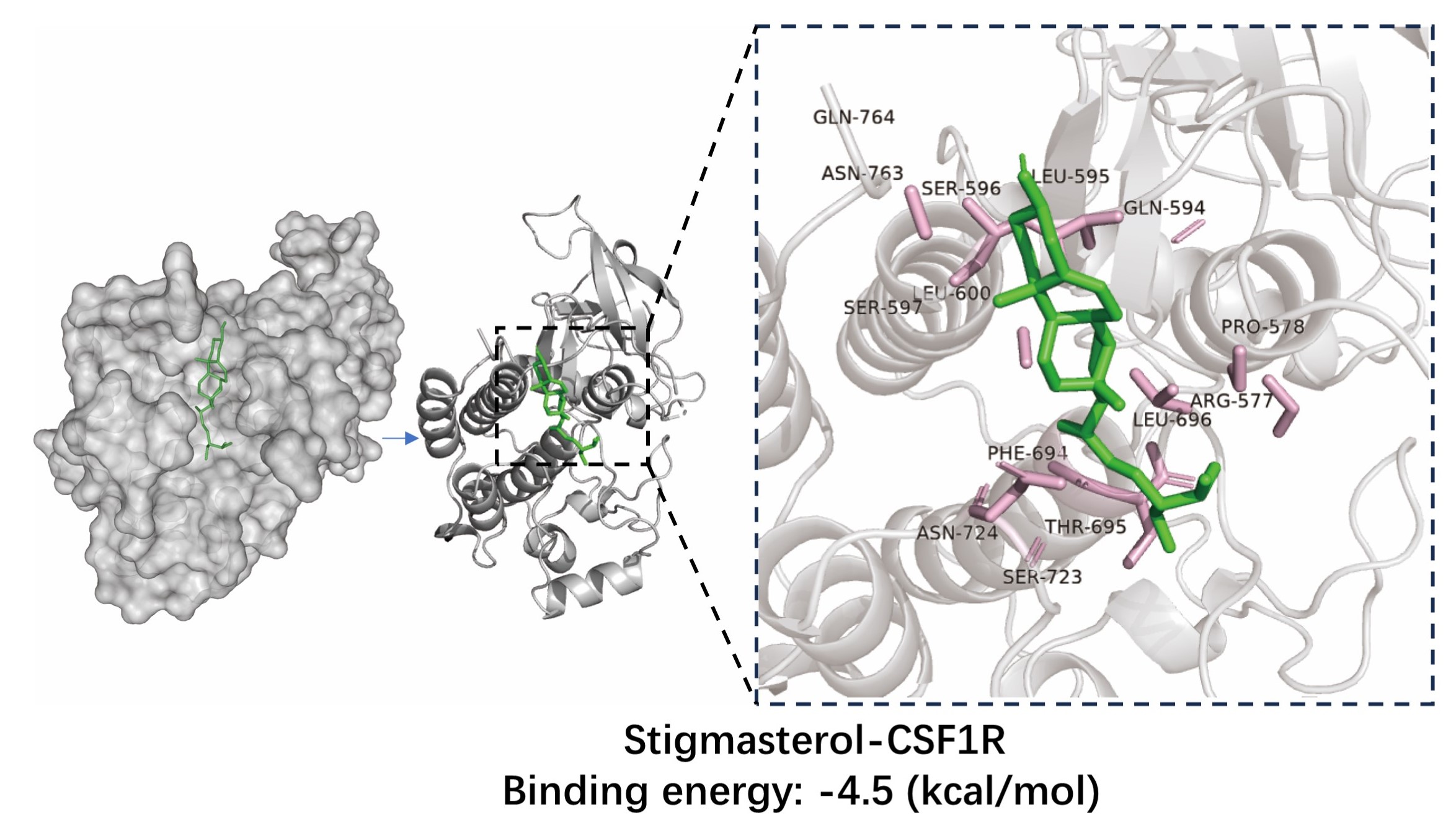

Supplement: Supplementary file 2 — Figure S2. [file CNS-30-e14657-s003.jpg]

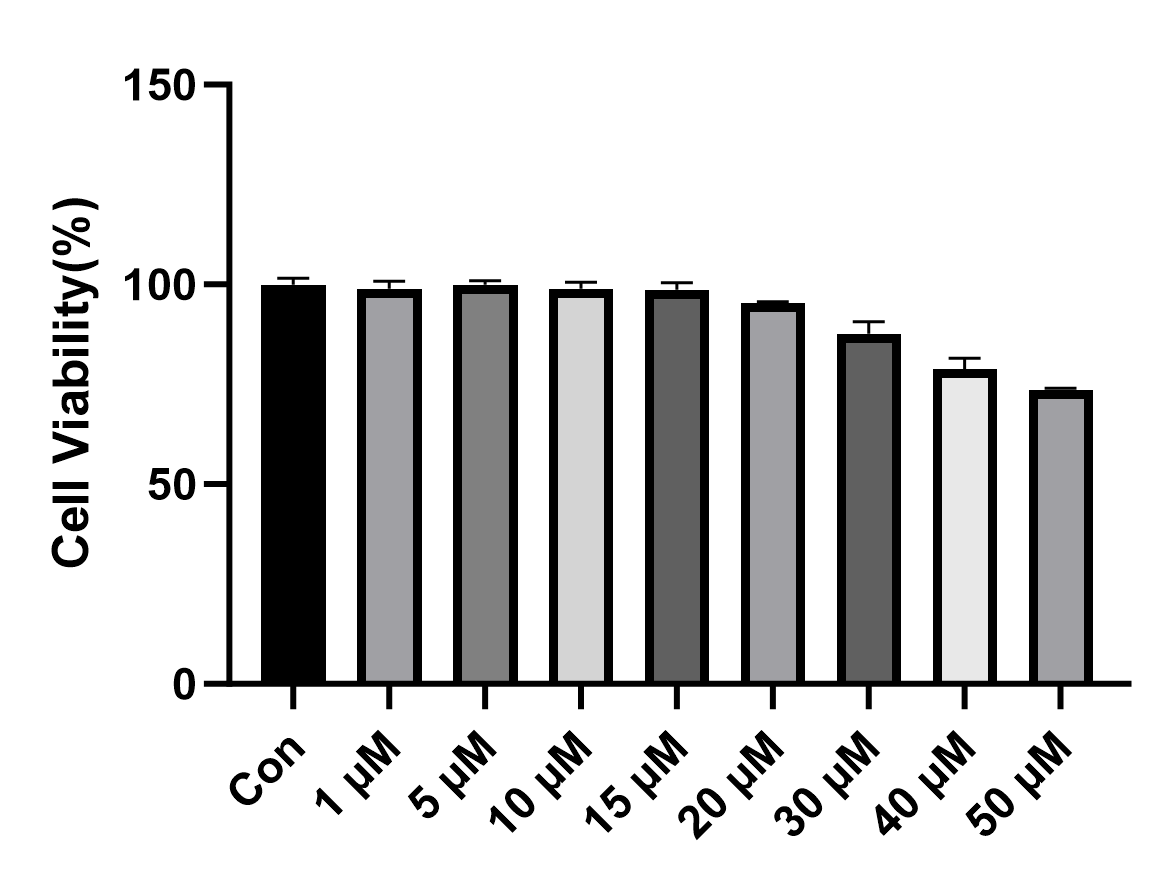

Supplement: Supplementary file 3 — Figure S3. [file CNS-30-e14657-s001.tif]
